# Urban villages as transfer stations for dengue fever epidemic: A case study in the Guangzhou, China

**DOI:** 10.1371/journal.pntd.0007350

**Published:** 2019-04-25

**Authors:** Hongyan Ren, Wei Wu, Tiegang Li, Zhicong Yang

**Affiliations:** 1 State Key Laboratory of Resources and Environmental Information System, Institute of Geographic Sciences and Natural Resources Research, Chinese Academy of Sciences, Beijing, China; 2 College of Geographical Science, Fujian Normal University, Fuzhou, China; 3 Department of Infectious Diseases, Guangzhou Center for Disease Control and Prevention, Guangzhou, People’s Republic of China; University of Washington, UNITED STATES

## Abstract

**Background:**

Numerous urban villages (UVs) and frequent infectious disease outbreaks are major environmental and public health concerns in highly urbanized regions, especially in developing countries. However, the spatial and quantitative associations between UVs and infections remain little understood on a fine scale.

**Methodology and principal findings:**

In this study, the relationships between reported dengue fever (DF) epidemics during 2012–2017, gross domestic product (GDP), the traffic system (road density, bus and/or subway stations), and UVs derived from high-resolution remotely sensed imagery in the central area of Guangzhou, were explored using geographically weighted regression (GWR) models based on a 1 km × 1 km grid scale. Accounting for 16.53%–18.07% of residential area and 16.84%–18.02% of population, UVs possessed 28.55%–38.24% of total reported DF cases in the core area of Guangzhou. The density of DF cases and the DF incidence rates in UVs were 1.81–3.13 and 1.82–3.06 times of that of normal construction land. Approximately 90% of the total cases were concentrated in the UVs and their buffering zones of radius ranged from 0 to 500 m. Significantly positive associations were observed between gridded DF incidence rates and UV area (*r* = 0.33, P = 0.000), the number of bus stops (*r* = 0.49, P = 0.000) and subway stations (*r* = 0.27, P = 0.000), and road density (*r* = 0.39, P = 0.000). About 60% of spatial variations in the gridded DF incidence rates were interpreted by the different variables of GDP, UVs, and bus stops integrated in GWR models.

**Conclusions:**

UVs likely acted as special transfer stations, receiving and/or exporting DF cases during epidemics. This work increases our understanding of the influences of UVs on vector-borne diseases in highly urbanized areas, supplying valuable clues to local authorities making targeted interventions for the prevention and control of DF epidemics.

## Author summary

Due to the rapid urbanization of China, many villages in the urban fringe are enveloped by ever-expanding cities and become so-called urban villages (UVs). UVs are widely distributed in not only the Guangzhou core areas but also the other cities in the highly urbanized region of China (e.g., Shenzhen, Wuhan). UVs are commonly featured by poor sanitation, overcrowding population, absent infrastructure, and some environmental pollution due to the development is neither authorized nor planned, resulting in a high environmental suitability for some vectors (e.g., *Aedes albopictus*), as well as the vetor-borne diseases (i.e., dengue fever) in these regions. In this study, we demonstrated that UVs may serve as transfer stations for the transmission of DF epidemic in the regions with developed transportation, higher GDP and dense population. This is manifested as that the rates of DF incidences were significantly positively associated with UV area. Furthermore, the density of DF cases and the DF incidence rates in UVs were 1.81–3.13 and 1.82–3.06 times of that of normal construction land and about 90% of the total DF cases were concentrated in 500m radius of UVs’ buffers. And the aggregation effects of UVs on this epidemic in the central region were obviously affected by public traffic conditions at the grid level. This study is the first quantitative analysis of the spatial relationship between UVs, public transportation, road density, population density, GDP and DF epidemics, which will provide a useful reference for accurately preventing and controlling DF epidemic in urban regions with numerous UVs.

## Introduction

Dengue fever (DF) is a febrile illness caused by the dengue virus, which is further classified into four serotypes (Dengue virus 1–4), and transmitted by *Aedes aegypti* and *Aedes albopictus* mosquitoes [1]. Dengue is the most prevalent mosquito-borne viral infection of humans in the tropical and subtropical regions of the world. Approximately 2 to 4 billon people are at risk of contracting dengue virus every year, resulting in nearly 100 million confirmed cases and causing ongoing wide concern [2–5]. After the founding of the People’s Republic of China, DF was eliminated in mainland China. However, increased openness and movement across borders have resulted in a recent revival of this tropical infectious disease, which is an imported epidemic to China [6, 7]. Approximately 94% of indigenous cases in mainland China were reported from Guangdong Province, and 83% of these cases were in Guangzhou City [8], following an unprecedented dengue outbreak in Guangzhou in 2014 that has attracted the attention of relevant researchers.

Both domestic and foreign scholars have carried out a considerable amount of research into DF epidemics, including into factors affecting the spread and prevalence of the disease and the corresponding prevention and control measures [9–13]. These studies have shown that the population, transportation, and living environment have undergone tremendous changes due to rapid urbanization, which in turn has led to changing DF transmission characteristics. In addition to some important natural environmental factors (e.g., temperature, precipitation) [9–11], social and economic factors, such as population distribution and density, land urbanization level, and road network density will have an important impact on the temporal and spatial patterns of DF epidemics [12, 13]. Furthermore, the presence of infected people may accelerate the transmission of DF in regions with high population densities [14, 15].

Informal urban settlements in China are described as urban villages (UVs), unique areas of high population density. Urban space has undergone dramatic transformation and reconstruction during China’s rapid urbanization, which has caused a large number of rural villages that were originally on the edge of the city to be gradually surrounded or semi-enclosed by urban land [16–20]. A lack of overall planning and scientific management of UVs has resulted in a large number of irregular buildings scattered in urban areas, with subsequent poor sanitation, lack of infrastructure and serious environmental pollution [21, 22]. These characteristics of UVs, combined with their perennial humidity and relatively low temperature [23, 24], provides an ideal living environment for the breeding of *Aedes albopictus*, the sole vector of dengue transmission in Guangzhou. However, the current quantitative relationship between UVs and DF epidemics has received very little attention.

Moreover, different factors influencing the DF epidemic have a spatial scale effect. Most of the available research has been focused on analysis at a relatively large spatial scale, such as at regional and prefecture level [25–28], with a small number of studies gradually expanding to small scales, such as at county, township, and even community or regular grid scales [29, 30]. However, these small spatial scales are often the final nodes where prevention and control measures can produce practical effects, and more research into the factors influencing DF and its prevention is required at this scale.

Therefore, this study was based on high-resolution remotely sensed imagery extraction of UVs in the central areas of Guangzhou, using epidemiological statistical methods and spatial analysis to further analyze the spatial relationship between UVs, public transportation, road density, population density, gross domestic product (GDP) and the DF epidemic according to a 1 km×1 km grid scale. The aim was to provide effective guidance for relevant government departments making targeted prevention and control measures on the DF epidemics in urban regions with numerous UVs.

## Materials and methods

### Study area

**Fig 1:**
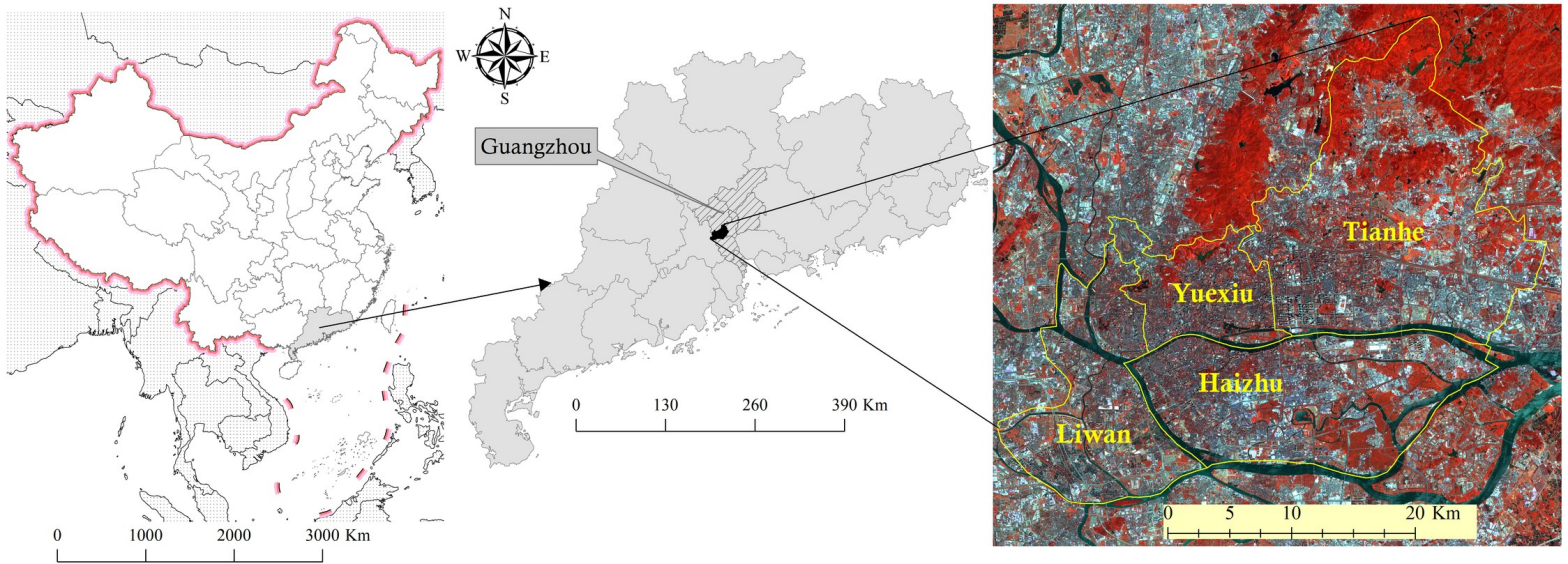
Study areas and GF-2 satellite data coverage of the central areas in Guangzhou.

The study area was located in the central areas of Guangzhou (113° 23'–113° 36'E, 23° 08'–23° 14'N) and included the four districts of Liwan, YueXiu, Haizhu, and Tianhe. The regional location is shown in Fig 1. The central area of the study was a highly urbanized area of Guangzhou, with an urbanization level of 100% and a total area of 279.63km^2^. This area has a population of 5.24 million permanent residents according to the 2017 Guangzhou Statistical Yearbook and is also the economic center of Guangzhou. In 2017, the GDP of the central area reached US$151.73 billion [31]. The characteristics of its subtropical monsoon climate are obvious: warm and rainy, enough light and heat, an annual average temperature of 21–23°C, and an average annual precipitation of 1800 mm. These suitable natural and social environmental conditions are favorable to the growth of *Aedes albopictus* and to the transmission of DENV, making it a high-risk area for DF [32]. In addition, Guangzhou, in particular the central area, has a large number of UVs due to rapid urbanization over the past decades [33].

### Data collection and processing

**Fig 2:**
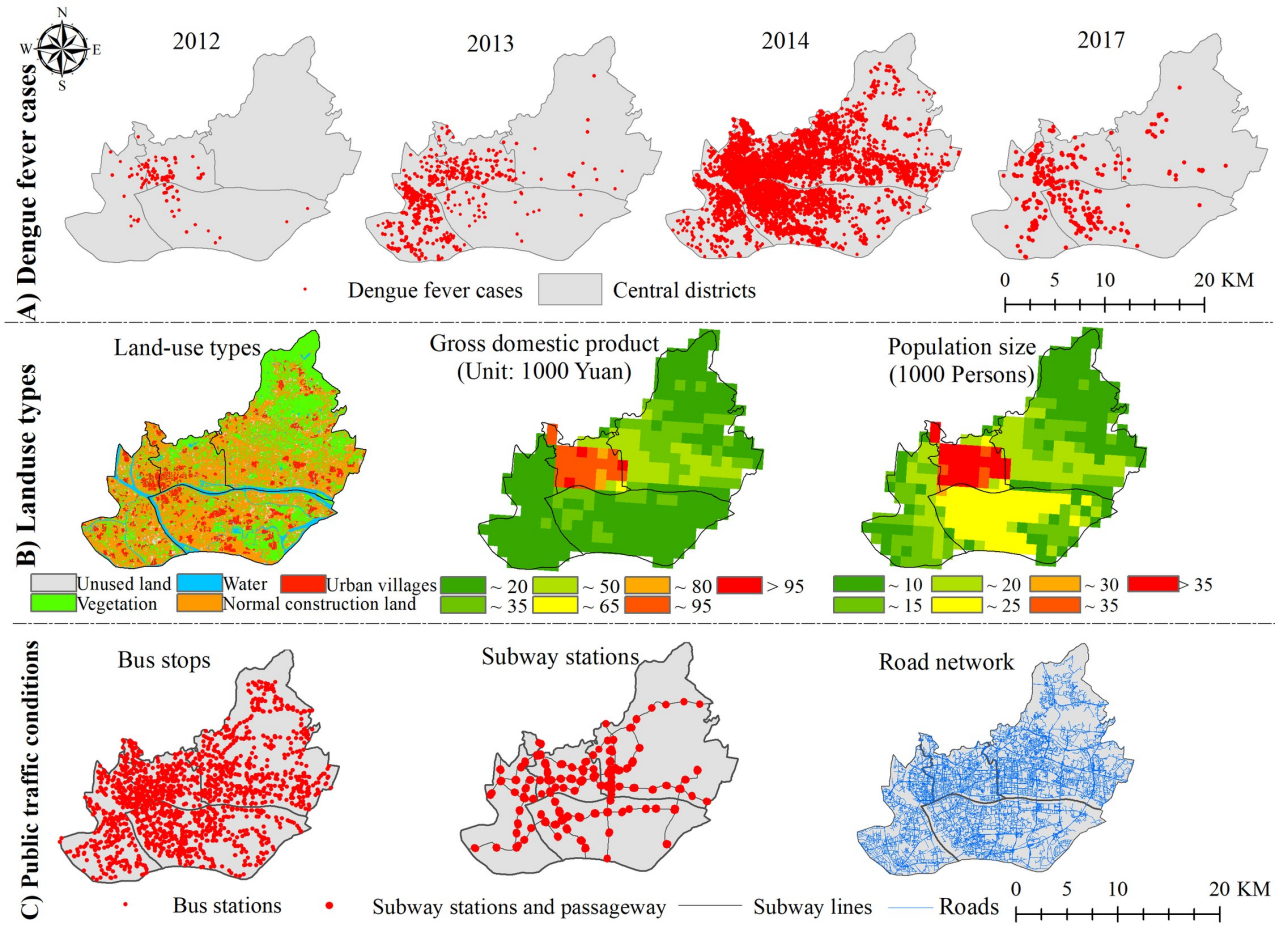
Spatial distribution and temporal variations of different data. (A): DF cases in 2012~2014 and 2017; (B): Spatial distribution of land use types, GDP and population density; (C): bus stations, subway stations and road network in 2017.

DF is a notifiable disease in China which means that, once diagnosed, cases must be reported to the web-based National Notifiable Disease Reporting Information System (NIDRIS) within 24 h [34]. The DF case information includes age, sex, address, and time of onset. The DF epidemic data for this study were obtained from the Guangzhou Center for Disease Control and Prevention, and included DF case data from 2012–2014 and from 2017. The targeted DF cases in our study included clinically diagnosed (based on clinical manifestations and epidemiologic exposure history) or laboratory-confirmed cases (“clinically diagnosed cases presenting with any of the following lab test results relating to DF: a 4-fold increase in specific IgG antibody titer, positive on a PCR test or viral isolation and identification test”). The address information of the confirmed cases, after desensitization, was used in conjunction with geocoding (http://www.gpsspg.com/xGeocoding/) and coordinate deviation correction to produce case data for a spatial point layer using ArcGIS 10.3 (ESRI, Redlands, CA, USA) software (Fig 2A). In 2014, DF cases in the Guangzhou region reached a peak, with a total of 36 344 cases reported, of which 18 350 were from the central area, accounting for 50.49% of the entire Guangzhou city. Moreover, the ratio of the total DF cases in 2012–2017 to the population in 2015 was calculated so as to indicate the DF incidence rates during the study period on the 1 km × 1 km grid scale.

With consideration of the high degree of urbanization of the study area, land use types for 2012 and 2017 was divided into five categories: normal construction land (NCL), UV, water, vegetation and unused land. NCL and UVs are collectively referred to herein as construction land (CL) (Fig 2B). A total of 206 sample points were randomly selected to verify the classification results for 2012 and 2017. The overall accuracy and the Kappa coefficients for 2012 and 2017 were 82.67%, 0.802 and 87.40%, 0.851, respectively. As far as the producer’s and user’s accuracy were concerned, the UVs in 2017 possessed slightly lower accuracies (87.8% and 87.8%) than those of water (88.2% and 93.8%), and roads (90.0% and 87.1%), although the omission and commission of UVs had been appropriately controlled by the texture selection procedure. In a word, the present extraction accuracy can meet the requirements for further analysis. Detailed information about the retrieval of land-use types can be found in an earlier study [24].

The public transport system (bus stops, subway stations) greatly facilitates the travel of people living in the central area. Population density and GDP data in 2015 were collected to indicate population and economic status. The public transport, population density, GDP and road network vector data in this study (Fig 2B and 2C) were all obtained from the Resources and Environment Science Data Center (RESDC, http://www.resdc.cn). Road density was generated from road network vector data, including all roads in the central region of Guangzhou (highways, national ways, county roads, town roads, etc.), and it was the ratio of the length of road in each unit (grid) to the corresponding unit’s area.

### Spatial analysis of the DF epidemic

Spatial autocorrelation analyses are frequently utilized to explore the spatial patterns of incidence or mortality in terms of Moran’s I with z-score and/or *p*-value because of their high statistical power [35–37]. Moran’s I is produced by standardizing spatial autocovariance by the data variance using a measure of the connectivity of the data [38]. Generally, Moran’s I value ranges from −1 to 1 and a high positive Moran’s I value with larger z-score and/or appropriate *p*-value represents a tendency towards clustering, which means that adjacent units have similar incidence rates, whereas a low negative value indicates a tendency towards dispersal, which means that units with high incidence rates lie next to units with low incidence rates.

In addition, the choice of spatial scale is the basis of spatial analysis. In many studies of infectious disease epidemiology, basic geographic units such as districts or townships/streets are often disturbed by changing administrative divisions, and the creation of regular spatial grids can effectively avoid this phenomenon [39]. With reference to our previous research work [29, 30, 39], a spatial gridded unit of 1 km × 1 km was used as the spatial unit in this study, and we analyzed the spatial autocorrelation degree of the DF epidemic at this grid scale.

### Geographically weighted regression modeling

In view of the spatiotemporal heterogeneity of DF incidence rates, the DF epidemic may be affected by its potential influencing factors in different ways and to various degrees, which is appropriate to analyze using a geographically weighted regression (GWR) model. As an extension of the traditional multiple linear regression (i.e., ordinary least square, OLS), a GWR model embeds the attributes’ spatial location into the regression parameter, yielding a local regression together with local estimates of regression coefficients[40]. The local estimation of the parameters with GWR is expressed by Eq (1) as below:

$${\text{y}}_{i}=\beta_{0}\left({\text{u}}_{i},{\text{v}}_{i}\right)+\sum_{k=1}^{n}\beta_{ik}\left({\text{u}}_{i},{\text{v}}_{i}\right){\text{x}}_{ik}+\varepsilon_{i}\;\;\;\;\;\;\;\;\;(i=1,\;2,\ldots ,\;\;\;\text{m)}$$ (1)

where *i* = 1, 2,…, m denotes the number of spatial units in the central area of Guangzhou; $y_{i}$ is the dependent variable (the DF incidence rates during 2012–2017) at location *i*; independent variable $x_{ik}$ is the value of the *k* parameter at location *i, $x_{ik}$* referred to the value of an affecting factor *k* (such as land use types, GDP, and so on) at spatial unit *i*, which is specific for every spatial unit; $\left(u_{i},v_{i}\right)$ is the position coordinate of the sample point; $\beta_{0}$ is the intercept; $\beta_{ik}$ is the correlation coefficient for the independent predictor variable $x_{ik}$, which is to be estimated; and $\varepsilon_{i}$ represents random error. During the GWR modeling, the most important parameter, named as bandwidth, that controls the degree of smoothing in the model was chosen by selecting the method of the corrected Akaike Information Criterion (AICc). Then, every spatial unit has a set of specific parameters to reflect the relationship between dengue fever incidence rate and influencing factors. In particular, the variance inflation factor (VIF) had also been employed in this study to test the collinearity among these independent variables integrated in the models, since these selected explanatory variables likely correlated with each other. Finally, all the parameters derived from both GWR and OLS will be compared in terms of the values of AICc, Sigma (i.e, residual standard deviation), VIF, and adjusted R^2^, on which the performance of these models could be evaluated. At the same time, the spatial autocorrelation analysis on the standardized residual (StdResid) values of these models was further employed to evaluate the explanatory performances (e.g., spatial stability) of the OLS and GWR models. The Moran’s I values close to zero indicated that there is no spatial autocorrelation of the StdResid values, and the results would be more reliable and then recommended for subsequent analysis.

By means of the exploratory regression tool, twenty-five OLS and GWR models with higher Adjusted R^2^ and lower values of AICc, Sigma, and VIF were recommended. Integrating various combinations of influencing factors in the OLS/GWR models with the lowest AICc and Sigma, the highest adjusted R^2^, and lower VIF than 7.5, eleven univariate models and fourteen multivariate models were respectively conducted for the comparison between CL, UV/NCL, water, vegetation, public traffic, road density, population density and GDP grouped factors.

In addition, we created a series of buffering zones with increasing radii based on the boundaries of the UVs, in which the incidence rates, the proportion of the DF cases, as well as its growth rates, in different years in each buffering zone were counted. Meanwhile, Pearson correlation analysis was applied to explore the relationships between DF incidences and all of the potential variables (UVs, population density, GDP, bus stops, subway stations, and road density) at the significance level of 0.05 and 0.01, by which some appropriate potential variables could be accordingly considered into the GWR models.

All of the above spatial analysis and modeling were completed in ArcGIS 10.3 software (ESRI, Redlands, CA, USA). Typical correlation analysis was achieved using SPSS 19.0 (SPSS Inc., Chicago, IL, USA).

## Results

### Spatial distribution of UVs

**Fig 3:**
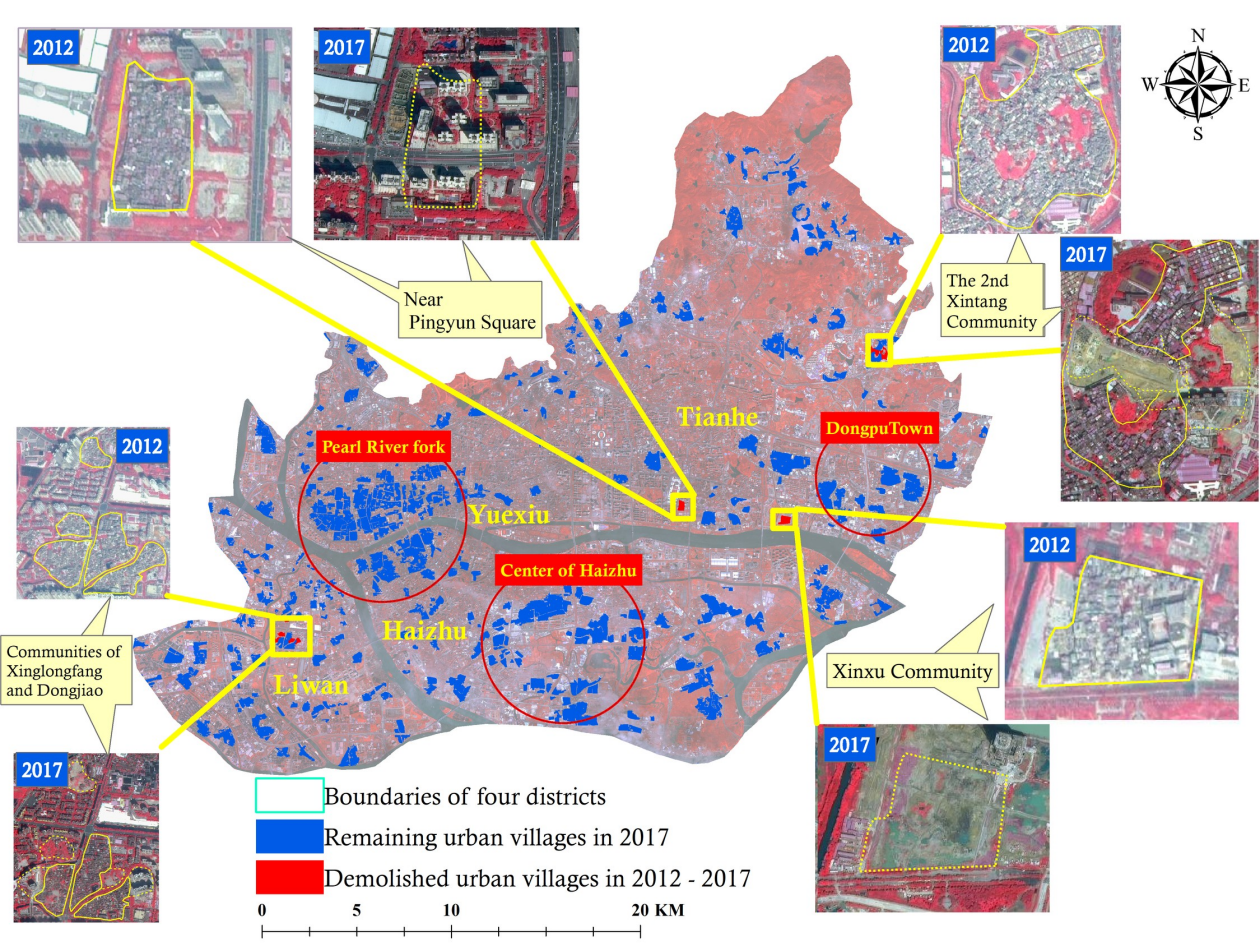
Spatial distribution of UVs across the central districts in Guangzhou during 2012–2017.

Land-use types across the central region, including Liwan, Haizhu, Yuexiu, and Tianhe districts in Guangzhou City, typically featured impervious surfaces (i.e., NCL and UVs) according to their dominant area percentage (53.80% in 2012 and 58.12% in 2017) (S1 Table). Among these four central regions, in 2012, Haizhu District had the largest area of UV (10.91 km^2^) and Yuexiu, Liwan, and Tianhe districts had 7.98, 4.06, and 8.80 km^2^ of UV. There were more than 450 UVs with areas varying from less than 0.001 km^2^ to 0.87 km^2^. As a result of urban renewal, the total area of UV decreased from 31.75 km^2^ (2012) to 31.38 km^2^ (2017), with a clear reduction in Liwan (about 0.12 km^2^ in Xinglongfang and Dongjiao communities) and Tianhe (0.25 km^2^ or so near Pingyun Square, the Second Xintang communities, and Xinxu communities) in particular, as shown in Fig 3. Since there were so fewer changes of UVs during 2012–2017, the data of land-use information in 2012 was chosen for subsequent analysis.

In the region, the Pearl River fork zone across Yuexiu, Liwan, and Haizhu was the most typically spatially clustered with UVs. Dongpu Town in Tianhe and the central zone of Haizhu were the other two representative zones. Unused land and water area decreased by 16.21 km^2^ and 1.95 km^2^, respectively, between 2012 and 2017, while vegetation and NCL increased by 4.03 km^2^ and 14.50 km^2^, respectively. In summary, the central area of Guangzhou City mainly featured impervious surfaces, especially many widely distributed UVs.

### Temporal and spatial distribution of the DF epidemic

**Fig 4:**
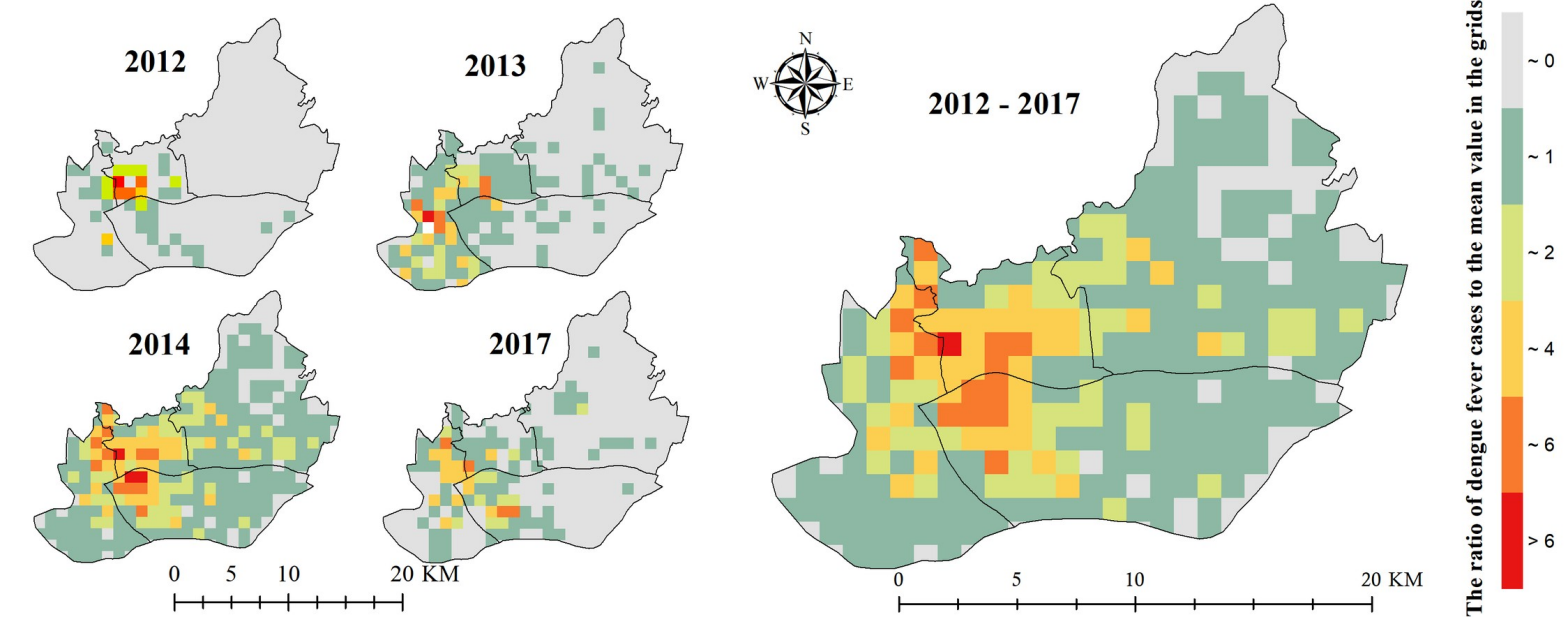
Spatial distribution of the gridded DF epidemic in 2012~ 2017. This is the spatial distribution of the ratio of DF cases in each infected unit to the mean value of all the infected units.

There were a total of 20 059 local DF cases reported in the central districts, which accounted for half of the total cases in the whole Guangzhou City during the study period. Meanwhile, the distribution of DF was spatially different across this typical region. The ratio of DF cases in each infected unit to the mean value of all the infected units (RDM) varied spatially on the 1 km × 1 km scale (close to the largest UV area) in 2012, 2013, 2014, and 2017. However, the units with high RDM were mainly located around the Pearl River fork between Yuexiu, Liwan, and Haizhu districts (Fig 4). Meanwhile, the DF epidemic during these four years was remarkably spatially clustered according to the spatial autocorrelation indices (0.174 < Moran’s I < 0.673, 6.398 < z-score < 16.930, *p*-value < 0.001; S2 Table). These results obviously showed that the DF epidemic in the central region was spatially featured on the grid scale.

### The aggregation effect of UVs on the DF epidemic

Between 2012 and 2017, DF patterns in the UVs differed from those of the NCL areas. As shown in Table 1, 61.76%– 71.45% of total DF cases in the CL zones were distributed in the NCL zones with proportionally larger areas (81.93% in 2012 and 83.47% in 2017) across the central four districts. By comparison, the rest (28.55%– 38.24%) were distributed in UVs with proportionally smaller areas (18.07% in 2012 and 16.53% in 2017). Meanwhile, population density (persons per km^2^) in the UVs was slightly higher than that of NCL in 2012 and 2017, although the population size of NCL is 4.55–4.94 times that of the UVs. As a result, the density of DF cases and the DF incidence rates in UVs were respectively 1.81–3.13 and 1.82–3.06 times that of NCL between 2012 and 2017. It can be clearly seen that UVs possessed higher values of DF cases density, incidence rates, and population density in the central region of Guangzhou City. In other words, DF cases were more likely to be found in UV areas.

**Table 1:** Comparison of DF epidemics and population in different CL types (NCL and UVs).

Year	Proportion of DF cases		Density (cases per km^2^)		Population density (person per km^2^)		DF incidence rates(cases / 10^5^)	
	NCL	UVs	NCL	UVs	NCL	UVs	NCL	UVs
2012	64.91%	35.09%	0.51	1.26	17732.83	18331.08	3.17	7.81
2013	62.64%	37.36%	3.96	10.71	/	/	27.04	73.36
2014	71.45%	28.55%	78.74	142.65	/	/	529.86	963.26
2017	61.76%	38.24%	1.77	5.54	17285.83	18386.52	12.09	36.95
Total	70.69%	29.31%	85.17	160.09	/	/	552.69	1086.71

The sizes of the DF epidemic were associated with the UVs’ area. In the UVs with recorded DF cases, the number of DF cases (the total in the study period) was strongly associated with acreage of these UVs (*r* = 0.45, *P* = 0.015), as shown in Table 2. Similarly, on the grid scale, the counts of DF cases were significantly correlated with the gridded UVs acreage (*r* = 0.33, *P* = 0.000) in the infected units.

**Table 2:** Correlation coefficients between DF incidence rates and UVs, pop density, GDP, traffic conditions^^^.

	Acreage of UVs	Pop density^○^	GDP^○^	UVs’ area^○^	Bus stops^○^	Subway stations^○^	Road density^○^
DF incidence rates^a^	0.45*	0.17**	0.10**	0.33**	0.49**	0.27**	0.39**
DF incidence rates^b^	/	/	/	0.24**	0.22**	0.33**	0.31**
DF incidence rates^c^	/	/	/	/	0.43**	0.27**	0.38**

Note:

^ denotes that the degrees of freedom for infected UVs and infected units were respectively 333 and 290.

^○^ indicates that the research unit is the 1km×1km grid scale.

* This value is significant at the level of 0.05.

** This value is significant at the level of 0.01.

a is all variable correlation analysis.

b is partial correlation coefficients between incidence rates and UV area while respectively controlling the traffic conditions.

c is partial correlation analysis between incidence rates and traffic conditions while controlling UV area.

**Fig 5:**
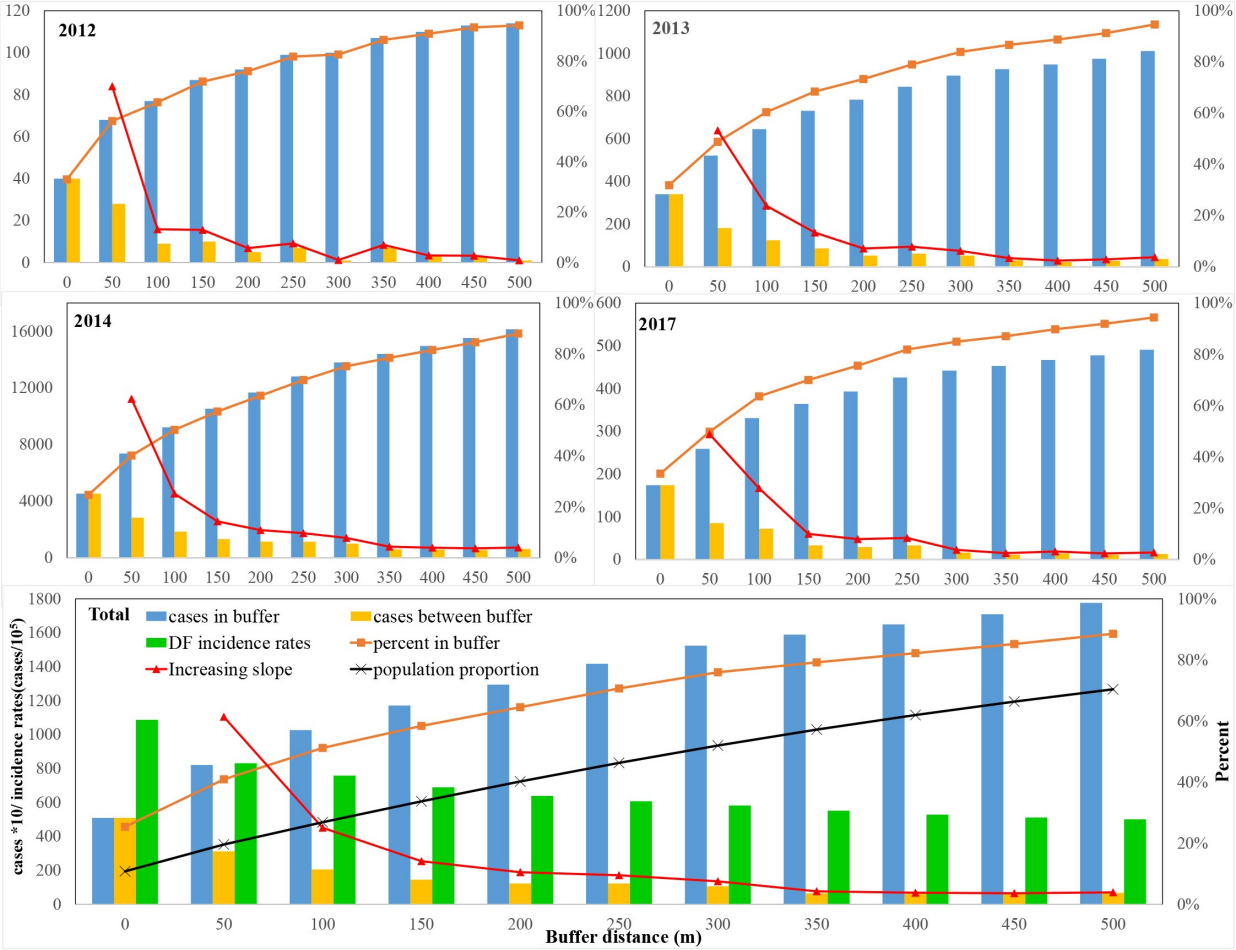
The aggregation effect of UVs on the DF cases across the central region in Guangzhou City. AVG is the average results of four years. Cases in buffer represent that DF cases count in different buffer radius. Cases between buffer indicate the number of DF cases between different buffer zones. Percent in buffer indicate the proportion of DF cases in different buffer radius. Increasing slope indicate the increasing rates of DF cases in different buffer radius.

In addition, the regions surrounding UVs were obviously influenced by the DF epidemic in the UVs with DF cases. Along with the radius of buffers increasing, accumulated DF case count in regions including the UVs with DF cases and their surrounding buffering zones showed an ascending trend (Fig 5). Until the radius of the buffer zones was 500m, about 90% of the total DF cases were concentrated in these regions (i.e., UVs and buffer zones). In comparison, newly included DF cases in the extended buffers per 50 m (i.e., increasing slope) displayed clear decreasing trends, especially in the first two buffers (50 m and 100 m). This decline was alleviated in the 200 m buffers. Meanwhile, the incidence rates of DF in the buffering zones gradually decreased, although there was larger and larger population proportion due to the increasing buffering distances. These results illustrated that UVs posed an obvious aggregation effect on the DF epidemic across the central region in Guangzhou City.

### Effects of transportation and UV area on the DF epidemic

Advanced traffic conditions, especially public transportation systems (such as bus services and subway lines) facilitate contact among people living in UVs. DF case density in the units (n = 272) with either bus stops or subway stations was much higher (73.21 cases per km^2^) than those without any bus stops or subway stations (7.74 cases per km^2^) (n = 19). Moreover, as given in Table 2, the gridded DF incidence rates were significantly positively associated with UV area (*r* = 0.33, P = 0.000), number of bus stops (*r* = 0.49, P = 0.000) and subway stations (*r* = 0.27, P = 0.000), and road density (*r* = 0.39, P = 0.000).

In comparison, the partial correlation coefficient between the gridded DF incidence rates and UV area individually decreased from 0.33 to 0.24 when traffic conditions were controlled for (Table 2). These partial correlation coefficients between DF incidence rates and traffic conditions (i.e., the number of bus stops, subway stations, all stops, and road density) were slightly decreased to 0.43, 0.27, 0.41, and 0.38, respectively, when UV area was controlled for. Among them, bus stops were the most suitable indicator of traffic conditions because of their high correlation coefficients. These results indicated that the aggregation effects on the gridded DF epidemic across the central region were heavily influenced by the traffic system, especially the presence of bus stops.

### Spatial modeling of DF epidemics

According to the adjusted R^2^, AICc and Sigma values (Table 3), spatial variations in the gridded DF epidemic in the central region of Guangzhou City were appropriately explained by the GWR/OLS models, which employed each influencing factor or their various combinations. In comparison, the comprehensive explanatory performance of the GWR models was much better than that of the OLS models due to the higher adjusted R^2^, lower AICc and Sigma values. About 46% or more spatial variation was interpreted by the univariate GWR models. Among the potential influencing factors derived from the univariate GWR models, bus stops and UVs possessed relatively higher adjusted R^2^, lower Sigma and AICc values. However, population density possessed a relatively lower adjusted R^2^, higher Sigma and AICc values.

**Table 3:** GWR and OLS modeling of DF incidence rates and different variables in the central region.

Models	Independent variables	GWR			OLS			VIF
		AICc	Adj- R^2^	Sigma	AICc	Adj- R^2^	Sigma	
Uni1	Bus stops	5340.50	0.55	285.34	5451.95	0.34	345.48	-
Uni2	Subway stations	5391.17	0.50	300.17	5565.04	0.11	401.69	-
Uni3	Road density	5341.64	0.54	289.47	5483.28	0.28	360.21	-
Uni4	Gross domestic production (GDP)	5403.32	0.48	307.38	5593.32	0.04	417.14	-
Uni5	Construction land (CL)	5338.99	0.54	288.27	5482.26	0.29	359.72	-
Uni6	Urban villages (UVs)	5368.70	0.55	287.47	5535.84	0.18	386.36	-
Uni7	Normal construction land (NCL)	5385.21	0.49	305.09	5535.99	0.18	386.44	-
Uni8	Unused land (UL)	5417.63	0.46	314.42	5610.90	0.00	427.03	-
Uni9	Water	5401.58	0.48	307.68	5611.02	0.00	427.09	-
Uni10	Vegetation	5408.15	0.49	306.01	5580.17	0.08	409.89	-
Uni11	Population density (POP)	5412.26	0.47	311.05	5560.88	0.12	399.48	-
Com1	Bus stops; UVs	5331.99	0.58	276.89	5432.77	0.38	336.29	1.23
Com2	Bus stops; Road density	5344.52	0.54	290.32	5439.96	0.37	339.52	2.18
Com3	Bus stops; Subway stations	5352.45	0.55	286.50	5453.39	0.34	345.66	1.39
Com4	Bus stops; NCL	5364.79	0.52	296.93	5451.70	0.35	344.88	1.70
Com5	Bus stops; GDP	5323.48	0.56	283.19	5448.03	0.35	343.20	1.36
Com6	Bus stops; POP	5340.35	0.55	285.39	5453.74	0.34	345.82	1.66
Com7	Bus stops; UVs; Road density	5334.96	0.55	284.87	5421.47	0.40	330.79	<2.37
Com8	Bus stops; UVs; Subway stations	5344.61	0.56	274.12	5433.13	0.38	335.98	<1.69
**Com9**	**Bus stops; UVs; GDP**	5305.02	0.59	272.80	5429.72	0.39	334.45	<1.63
Com10	Bus stops; UVs; POP	5321.19	0.59	272.86	5434.04	0.37	336.38	<1.84
Com11	Bus stops; UVs; POP; Subway stations	5330.78	0.57	278.59	5434.05	0.38	335.92	< 2.18
Com12	Bus stops; UVs; GDP; POP	5337.36	0.53	293.25	5429.25	0.39	333.77	<4.82
Com13	Bus stops; UVs; POP; Road density; GDP;	5318.74	0.55	285.34	5410.83	0.42	325.22	<5.12
Com14	Bus stops; UVs; POP; Subway stations; Road density; GDP	5318.48	0.55	284.62	5411.91	0.42	325.22	< 5.12

Adj-R2: Adjusted R-Squared; AICc: the corrected Akaike's Information Criterion; VIF: Variance Inflation Factor.

**Fig 6:**
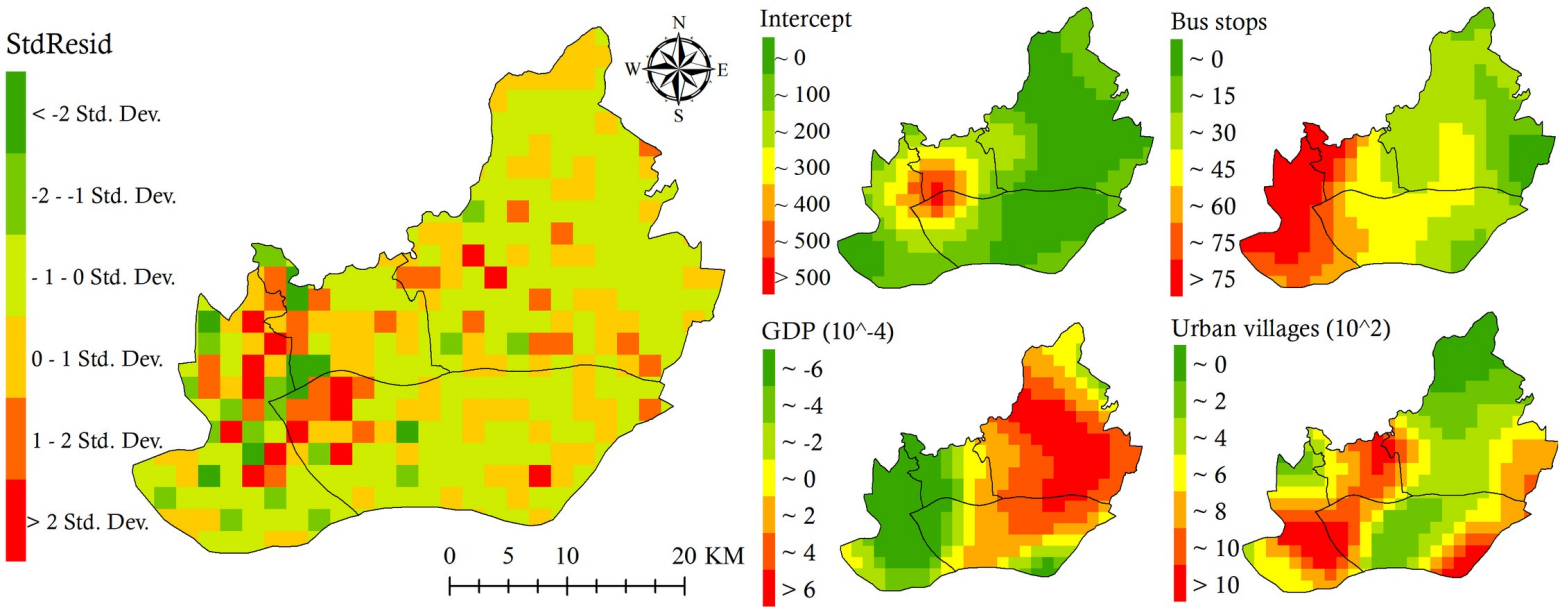
Standardized residual (StdResid) values and local coefficients of selected variables. This is derived from the GWR model with GDP, UVs and bus stops.

Similarly, the GWR model (Com 1) integrating bus stops and UVs performed the best among all of the bivariate models. When this model further integrated either GDP or population density that possessed relatively weaker explanatory ability, the performances were increased according to the rising adjusted R^2^ and declining values of AICc and Sigma in the GWR models (Com 9 and Com 10 in Table 3). In comparison, the other multivariate GWR models (Com 7, 8, and 11–14) had slightly weaker performances. In particular, the weakest performance was observed in the model (Com 12) even though the best bivariate GWR model (Com 1) further integrated both GDP and population density. And the reliability of its results quickly decayed because the potential risk of collinearity (VIF) had increased from 1.23 to 4.82 (Table 3). In all of the models, the GWR model (Com 9) integrating UVs, bus stops, and GDP possessed the highest adjusted R^2^ (0.59), the lowest AICc (5305.02), the lowest Sigma (272.80), and a lower VIF value (<1.63). Moreover, according to the spatially random patterns of the StdResid values (Moran’s I = 0.04, *p* = 0.26, Z-score = 1.12), the performance of this model was also spatially stable, although there were still a few units with absolute StdResid values > 2 (Fig 6).

In terms of the influence of the three variables (GDP, UVs, and bus stops) on the spatial variation of DF infection, these variables were spatially differentiated across the central region (Fig 6). The presence of bus stops was positively associated with the DF epidemics in most of the units, especially the Liwan district. The influences of GDP displayed spatial disparities. They were positive effect in most areas of Tianhe and Haizhu districts and negative effect in Liwan, Yuexiu, and near the Pearl River fork of Haizhu district. In comparison, UVs tended to have a greater association with DF, with more units with relative higher local coefficient values in Liwan, Yuexiu, Haizhu, and Tianhe districts, although the association tended to be weaker (less than 200) in the central Haizhu where there were relatively fewer DF cases (Fig 4), lower road density, less population density, and sparser public transportation stations (Fig 2). These results suggested that UVs were the most important factor in the spatial variations in the DF epidemic across the central region of Guangzhou.

## Discussion

Widely distributed urban settlements and serious DF epidemics are two major public concerns in Guangzhou City. In this study, we analyzed the spatial and quantitative relationship between the DF epidemic and UVs on a grid scale across the central region of Guangzhou. The interesting findings provide valuable clues to enable local environmental health authorities in targeted interventions in the prevention of this epidemic.

The size and spatial heterogeneity of the DF epidemics were probably associated with numerous UVs widely distributed across the study region with developed public transportation (e.g., many bus stops), good economic status, and a dense population. Previous studies across the entire joint Guangzhou-Foshan (GF) area have found that spatially clustered DF cases in this region are associated with its higher land urbanization level, population size, road density, and economic level (GDP per capita) [30, 41]. Our study found that this central region typically featured by not only impervious surfaces (including NCL and UVs) but also spatially differentiated DF epidemics on the 1 km × 1 km grid scale. Meanwhile, there was higher density of DF cases and incidence rates in UV areas than in NCL areas, and the DF epidemic was significantly positively associated with UVs’ acreage both at the grid scale and the UV level. There are two possible reasons for this. First, UVs, as a type of informal urban settlement, provide *Aedes albopictus* mosquitoes with a suitable environment for survival and breeding, featuring slightly lower land surface temperature than NCL areas [24], especially in the summer months. Second, the denser population in UVs and its flowing traits were two core impetuses of UVs’ influences on the DF epidemic. In particular, UVs have a high density of low-cost accommodation rented by migrant workers from local house owners, which provided local citizens with a steady source of revenue and resulted in a large floating population [21, 42]. This increases the probability of being bitten by mosquito vectors, causing rapid transmission of this disease. DF is also more frequent within a specific 200–300 m radius around UVs, which is probably determined by the mosquitoes’ maximum flying range of approximately 300 m [43, 44]. Thus, it can be seen that widely distributed UVs have an important influence on the DF epidemic, not only within the specific UV areas but also within their surrounding zones, crucially associated with the severity and obvious spatial disparities in DF incidence across the central region in Guangzhou City. We cautiously suggest that both UVs and their surrounding zones should receive considerable focus during DF epidemics.

A convenient public traffic system meets the commuting demands of local residents, including the floating population who reside in UVs but work outside of them. Earlier studies reported that traffic conditions impose important effects on DF transmission [13, 41, 45], similar to our findings that public transportation (the presence of bus stops in particular) was not only directly associated with the DF incidence rates on the grid scale, but also influenced the aggregation effect of UVs on the DF epidemic, especially in zones with numerous UVs. We cautiously speculate that DF cases in infected UVs or units (grids) may have been potential infection sources when they entered other zones via the public traffic system. In other words, these UVs or units/grids with infections presence likely acted as transfer stations (receiving and/or exporting DF cases) during DF transmission. In a sense, it can be supposed to some degree that the infected cases/patients would also act as the disease’s vector, since local residents are the other crucial element in the DF transmission and the flying distance of mosquitoes (*Aedes albopictus*) is far less than human mobility due to developed public transportation. Accordingly, we recommend that targeted and effective interventions should be implemented in zones with numerous UVs and public traffic stations across the central region in Guangzhou City during the DF epidemic.

Use of generalized additive models has revealed that associations between DF infection and GDP were nonlinear at the township level across the Pearl River Delta [13]. Our earlier investigation found that GDP had a weak but spatially differentiated correlation with DF infection across the GF area [29]. We found similar in this study, employing GWR models at a fine spatial scale (1 km × 1 km) and taking three spatially differentiated variables (UVs and bus stops as special influencing factors, as well as GDP) into consideration. GDP had a clear protective effect in the west zones (including in Liwan, most of Yuexiu, and west Haizhu) where there were more serious DF epidemics and larger areas of UV. This effect is likely related to the UVs in these areas being surrounded by NCL, with a higher economic status and the better promotion of public health services (e.g., education and publicity about hygiene) in these well-developed zones. On the contrary, GDP tended to be a risk factor for DF transmission in the east zones (i.e., most of Tianhe and northeast Haizhu) where there were relatively small DF outbreaks and many construction sites (categorized as unused land in this study), especially in the district of Tianhe, which is experiencing rapid economic development and urban construction. However, whether the positive correlation between GDP and DF incidence rates was related to the wide distribution of unused land needs further investigation. Nevertheless, our findings are sufficiently reasonable and detailed to infer that the influence of economic status on DF transmission was spatially differentiated. We advise that the protective influences of GDP on DF infection in Liwan and Yuexiu districts are further investigated to explore how its protective effects can be expanded to Tianhe and Haizhu districts with their growing economies.

There were some limitations to the study. First, many more important influential factors should be further explored and included in the GWR models to interpret much more spatial variation in the DF epidemic, since less than 60% was explained at present. In particular, an appropriate variable should be acquired to comprehensively reflect both public transportation and population density so as to further interpret the remained (40% or so) spatial variation in the current study. For this point, data derived from mobile devices, metro cards and/or bus cards could be used to capture information about mode of travel and the movement of local residents, both for confirmed and suspected DF cases. This is particularly relevant to the UVs, as the effects of the public transportation system and UVs on DF transmission could be further investigated and high intercept values (Fig 6) decreased. Second, reliable monitoring data on the vectors’ population or density in the study area should be continuously collected and then used fully for a further comprehensive analysis of the link between DF epidemic and all the influencing factors in the future. Finally, the time series of the DF case data and the spatiotemporally matched remote sensing images should be longer. This would enable better validation of the typical influence of UVs, as DF epidemics have periodically occurred in the central region of Guangzhou City, with rapid land urbanization, since the 2000s.

## Supporting information

S1 Table The transfer-matrix of different land-use types in the central region (km^2^). (DOC)

S2 Table Spatial autocorrelation analysis of the DF cases on the point and grid scale during 2012–2017. (DOCX)

S3 Table The matrix of correlation coefficients between all the selected variables on the grid scale. (DOCX)

## References

[1] Guzman MG, Harris E. Dengue. Lancet. 2014;385(9966):453–65. 10.1016/S0140-6736(14)60572-9 25230594

[2] Bhatt S, Gething PW, Brady OJ, Messina JP, Farlow AW, Moyes CL, et al The global distribution and burden of dengue. Nature. 2013; 496(7446):504–7. Epub 2013/04/09. 10.1038/nature12060 23563266 PMC3651993

[3] Gubler DJ. Dengue, Urbanization and Globalization: The Unholy Trinity of the 21st Century. International Journal of Infectious Diseases. 2012;16(4 Suppl):3–11. 10.2149/tmh.2011-S05 PMC3317603 22500131

[4] Organization WH. Global Strategy for Dengue Prevention and Control, 2012–2020 WHO, Geneva 2012: World Health Organization; 2012.

[5] Halstead SB. Dengue haemorrhagic fever—a public health problem and a field for research. Bulletin of the World Health Organization. 1980;58(1):1–21. 6966540 PMC2395896

[6] Wang C, Yang W, Fan J, Wang F, Jiang B, Liu Q. Spatial and Temporal Patterns of Dengue in Guangdong Province of China. Asia Pac J Public Health. 2013;27(2):844–53. 10.1177/1010539513477681 23467628

[7] Sang SW, Liu QY. Spatial and temporal analysis of indigenous dengue cases in Guangdong province during 2003–2012. Chinese Journal of Vector Biology & Control. 2015.

[8] Lai S, Huang Z, Zhou H, Anders KL, Perkins TA, Yin W, et al The changing epidemiology of dengue in China, 1990–2014: a descriptive analysis of 25 years of nationwide surveillance data. Bmc Medicine. 2015;13(1):100. 25925417 10.1186/s12916-015-0336-1 PMC4431043

[9] Zhang Y, Tao W, Liu K, Yao X, Yi L, Jing Q, et al Developing a Time Series Predictive Model for Dengue in Zhongshan, China Based on Weather and Guangzhou Dengue Surveillance Data. Plos Neglected Tropical Diseases. 2016;10(2):e0004473 10.1371/journal.pntd.0004473 26894570 PMC4764515

[10] Liang L, Lin H, Tian L, Yang W, Sun J, Liu Q. Time series analysis of dengue fever and weather in Guangzhou, China. Bmc Public Health. 2009;9(1):1–5. 19860867 10.1186/1471-2458-9-395 PMC2771015

[11] Zhu G, Liu J, Tan Q, Shi B. Inferring the Spatio-temporal Patterns of Dengue Transmission from Surveillance Data in Guangzhou, China. Plos Neglected Tropical Diseases. 2016;10(4):e0004633 10.1371/journal.pntd.0004633 27105350 PMC4841561

[12] Z L, W Y, Clements A, Williams G, S L, Zhou H, et al Spatiotemporal analysis of indigenous and imported dengue fever cases in Guangdong province, China. Bmc Infectious Diseases. 2012;12(1):132. 22691405 10.1186/1471-2334-12-132 PMC3412724

[13] Qi X, Wang Y, Li Y, Meng Y, Chen Q, Ma J, et al The Effects of Socioeconomic and Environmental Factors on the Incidence of Dengue Fever in the Pearl River Delta, China, 2013. Plos Neglected Tropical Diseases. 2015;9(10):e0004159 10.1371/journal.pntd.0004159 26506616 PMC4624777

[14] Wu PC, Lay JG, Guo HR, Lin CY, Lung SC, Su HJ. Higher temperature and urbanization affect the spatial patterns of dengue fever transmission in subtropical Taiwan. Science of the Total Environment. 2009;407(7):2224–33. 10.1016/j.scitotenv.2008.11.034 19157509

[15] Wen T, Lin M, Fang C. Population Movement and Vector-Borne Disease Transmission: Differentiating Spatial–Temporal Diffusion Patterns of Commuting and Noncommuting Dengue Cases. Annals of the Association of American Geographers. 2012;102(5):1026–37.

[16] CHUNG H. Building an image of Villages-in-the-City: A Clarification of China's Distinct Urban Spaces. International Journal of Urban and Regional Research. 2010;34(2):421–37. 10.1111/j.1468-2427.2010.00979.x 20827848

[17] Hao P, Hooimeijer P, Sliuzas R, Geertman S. What Drives the Spatial Development of Urban Villages in China? Urban Studies. 2013;50(16):3394–411.

[18] Taubenböck H, Kraff NJ. The physical face of slums: a structural comparison of slums in Mumbai, India, based on remotely sensed data. Journal of Housing & the Built Environment. 2014;29(1):15–38.

[19] Goebel A. Sustainable urban development? Low-cost housing challenges in South Africa. Habitat International. 2007;31(3):291–302.

[20] Hofmann P, Strobl J, Blaschke T, Kux H. Detecting informal settlements from QuickBird data in Rio de Janeiro using an object based approach: Springer Berlin Heidelberg; 2008 531–53 p.

[21] Liu H, Huang X, Wen D, Li J. The Use of Landscape Metrics and Transfer Learning to Explore Urban Villages in China. Remote Sensing. 2017;9(4).

[22] Huang X, Liu H, Zhang L. Spatiotemporal Detection and Analysis of Urban Villages in Mega City Regions of China Using High-Resolution Remotely Sensed Imagery. IEEE Transactions on Geoscience & Remote Sensing. 2015;53(7):3639–57. 10.1016/j.jenvman.2006.04.023

[23] Guo Q, Zou Z, Hongyong LI, Qiu G. Analysis on the Thermal Environment of Urban Village in Shenzhen. Ecology & Environmental Sciences. 2015.

[24] Wu W, Ren H, Yu M, Wang Z. Distinct Influences of Urban Villages on Urban Heat Islands: A Case Study in the Pearl River Delta, China. Int J Environ Res Public Health. 2018;15(8). Epub 2018/08/08. 10.3390/ijerph15081666 30082641 PMC6121422

[25] Peterson AT. Ecological niche modelling and understanding the geography of disease transmission. Veterinaria Italiana. 2007;43(43):393–400. 20422515

[26] Machado-Machado EA. Empirical mapping of suitability to dengue fever in Mexico using species distribution modeling. Applied Geography. 2012;33:82–93.

[27] Peterson AT, Martínezcampos C, Nakazawa Y, Martínezmeyer E. Time-specific ecological niche modeling predicts spatial dynamics of vector insects and human dengue cases. Transactions of the Royal Society of Tropical Medicine & Hygiene. 2005;99(9):647–55. 15979656 10.1016/j.trstmh.2005.02.004

[28] Cardoso-Leite R, Vilarinho AC, Novaes MC, Tonetto AF, Vilardi GC, Guillermo-Ferreira R. Recent and future environmental suitability to dengue fever in Brazil using species distribution model. Trans R Soc Trop Med Hyg. 2014;108(2):99–104. 10.1093/trstmh/trt115 24463584

[29] Li Q, Ren H, Zheng L, Cao W, Zhang A, Zhuang D, et al Ecological Niche Modeling Identifies Fine-Scale Areas at High Risk of Dengue Fever in the Pearl River Delta, China. International Journal of Environmental Research & Public Health. 2017;14(6):619. 10.3390/ijerph14060619 PMC5486305 28598355

[30] Ren H, Zheng L, Li Q, Yuan W, Lu L. Exploring Determinants of Spatial Variations in the Dengue Fever Epidemic Using Geographically Weighted Regression Model: A Case Study in the Joint Guangzhou-Foshan Area, China, 2014. International Journal of Environmental Research & Public Health. 2017;14(12):1518. 10.3390/ijerph14121518 PMC5750936 29211001

[31] Municipality. SBoG. Guangzhou Economic and Social Development Statistics Bulletin 2017. http://www.gdstats.gov.cn/tjzl/tjgb/.

[32] Wu JY, Lun ZR, James AA, Chen XG. Dengue fever in Mainland China. American Journal of Tropical Medicine & Hygiene. 2010;83(3):664. 20810836 10.4269/ajtmh.2010.09-0755 PMC2929067

[33] He JB. The Research on the Transformation of Urban Village in Pearl River Delta Developed Area Base on Case Study on the Xihu Village. Master’s Thesis,. 2013.

[34] Cheng Q, Jing Q, Spear RC, Marshall JM, Yang Z, Peng G. Climate and the Timing of Imported Cases as Determinants of the Dengue Outbreak in Guangzhou, 2014: Evidence from a Mathematical Model. Plos Neglected Tropical Diseases. 2016;10(2):e0004417 10.1371/journal.pntd.0004417 26863623 PMC4749339

[35] Walter SD. Assessing spatial patterns in disease rates. Statistics in Medicine. 1993;12(19–20):1885–94. 8272668 10.1002/sim.4780121914

[36] Antunes JL, Biazevic MG, de Araujo ME, Tomita NE, Chinellato LE, Narvai PC. Trends and spatial distribution of oral cancer mortality in Sao Paulo, Brazil, 1980–1998. Oral Oncology. 2001;37(4):345–50. 11337266 10.1016/s1368-8375(00)00113-5

[37] Schäfer T, Pritzkuleit R, Jeszenszky C, Malzahn J, Maier W, Günther KP, et al Trends and geographical variation of primary hip and knee joint replacement in Germany. Osteoarthritis & Cartilage. 2013;21(2):279–88. 23220558 10.1016/j.joca.2012.11.006

[38] Anselin L, Getis A. Spatial statistical analysis and geographic information systems. Annals of Regional Science. 1992;26(1):19–33.

[39] Ren H, Xu D, Shi X, Xu J, Zhuang D, Yang G. Characterisation of gastric cancer and its relation to environmental factors: a case study in Shenqiu County, China. International Journal of Environmental Health Research. 2016;26(1):1–10. 10.1080/09603123.2014.1003040 25608493

[40] Mcmillen DP. Geographically Weighted Regression: The Analysis of Spatially Varying Relationships. American Journal of Agricultural Economics. 2004;86(2):554–6.

[41] Li Q, Cao W, Ren H, Ji Z, Jiang H. Spatiotemporal responses of dengue fever transmission to the road network in an urban area. Acta Tropica. 2018;183. 10.1016/j.actatropica.2018.03.026 29608873

[42] Song Y, Zenou Y, Ding C. Let’s not throw the baby out with the bath water: The role of urban villages in housing rural migrants in China. Urban Studies. 2008;45(2):313–30.

[43] Liew C, Curtis CF. Horizontal and vertical dispersal of dengue vector mosquitoes, Aedes aegypti and Aedes albopictus, in Singapore2004. 351-60 p. 10.1111/j.0269-283X.2004.00517.x 15642001

[44] Maciel-de-Freitas R, Souza-Santos R, Codeco CT, Lourenco-de-Oliveira R. Influence of the spatial distribution of human hosts and large size containers on the dispersal of the mosquito Aedes aegypti within the first gonotrophic cycle. Med Vet Entomol. 2010;24(1):74–82. Epub 2010/04/10. 10.1111/j.1365-2915.2009.00851.x . 20377734

[45] Mahabir RS, Severson DW, Chadee DD. Impact of road networks on the distribution of dengue fever cases in Trinidad, West Indies. Acta Tropica. 2012;123(3):178–83. 10.1016/j.actatropica.2012.05.001 22609547

