# Supplementary material for: Urban villages as transfer stations for dengue fever epidemic: A case study in the Guangzhou, China
Source: PLoS Negl Trop Dis. 2019 Apr 25;13(4):e0007350. doi: 10.1371/journal.pntd.0007350 (PMC6504109; doi:10.1371/journal.pntd.0007350)
Supplement: S1 Table — (DOC) [file pntd.0007350.s001.doc]

**S1 Table.** The transfer-matrix of different land-use types in the central region of Guangzhou (km^2^).

| 2012  2017 | UV | NCL | Vegetation | Water | Unused land | Sum |
| --- | --- | --- | --- | --- | --- | --- |
| UV | 29.72 | 0.20 | 0.97 | 0.03 | 0.83 | 31.75 |
| NCL | 0.00 | 115.26 | 20.32 | 3.31 | 5.06 | 143.96 |
| Vegetation | 0.73 | 21.62 | 75.80 | 0.50 | 2.41 | 101.06 |
| Water | 0.13 | 4.46 | 1.15 | 17.49 | 0.19 | 23.42 |
| Unused land | 0.80 | 16.91 | 6.85 | 0.14 | 1.71 | 26.41 |
| Sum | 31.38 | 158.45 | 105.09 | 21.47 | 10.20 | 326.59 |

**Summary:** The total area of UV decreased from 31.75 km^2^ (2012) to 31.38 km^2^ (2017). Unused land and water area decreased by 16.21 km^2^ and 1.95 km^2^, respectively, between 2012 and 2017, while vegetation and NCL increased by 4.03 km^2^ and 14.50 km^2^, respectively.
