# Supplementary material for: Urban villages as transfer stations for dengue fever epidemic: A case study in the Guangzhou, China
Source: PLoS Negl Trop Dis. 2019 Apr 25;13(4):e0007350. doi: 10.1371/journal.pntd.0007350 (PMC6504109; doi:10.1371/journal.pntd.0007350)
Supplement: S2 Table — (DOCX) [file pntd.0007350.s002.docx]

**S2 Table**. Spatial autocorrelation analysis of the DF cases on the point and grid scale during 2012 - 2017. The spatial clustering of the DF epidemics at the grid scale is more significant and it is more suitable for spatial and temporal variation analysis.

| Year | Moran’s I | Z-score | P-Value | Scale |
| --- | --- | --- | --- | --- |
| 2012 | 0.3549 | 1.8940 | 0.0582 | Point |
| 2013 | 0.1884 | 9.1584 | 0.0000 |  |
| 2014 | 0.1210 | 49.7651 | 0.0000 |  |
| 2017 | 0.5913 | 13.7223 | 0.0000 |  |
| 2012-2014 and 2017 | 0.1219 | 53.7267 | 0.0000 |  |
| 2012 | 0.2814 | 24.3818 | 0.0000 | 0.3km×0.3km grid |
| 2013 | 0.2980 | 25.6642 | 0.0000 |  |
| 2014 | 0.3373 | 28.7111 | 0.0000 |  |
| 2017 | 0.2832 | 24.6260 | 0.0000 |  |
| 2012-2014 and 2017 | 0.3407 | 28.9957 | 0.0000 |  |
| 2012 | 0.3676 | 19.6331 | 0.0000 | 0.5km×0.5km grid |
| 2013 | 0.2909 | 15.5068 | 0.0000 |  |
| 2014 | 0.5191 | 26.6191 | 0.0000 |  |
| 2017 | 0.3192 | 16.7589 | 0.0000 |  |
| 2012-2014 and 2017 | 0.5164 | 26.4753 | 0.0000 |  |
| 2012 | 0.3721 | 12.9242 | 0.0000 | 0.8km×0.8km grid |
| 2013 | 0.4595 | 15.6155 | 0.0000 |  |
| 2014 | 0.6471 | 21.1883 | 0.0000 |  |
| 2017 | 0.4294 | 14.4988 | 0.0000 |  |
| 2012-2014 and 2017 | 0.6530 | 21.3785 | 0.0000 |  |
| 2012 | 0.4444 | 12.1766 | 0.0000 | 1km×1km grid |
| 2013 | 0.4651 | 12.2791 | 0.0000 |  |
| 2014 | 0.6706 | 15.0853 | 0.0000 |  |
| 2017 | 0.1741 | 6.3983 | 0.0000 |  |
| 2012-2014 and 2017 | 0.6728 | 16.9304 | 0.0000 |  |
