# Supplementary material for: Urban villages as transfer stations for dengue fever epidemic: A case study in the Guangzhou, China
Source: PLoS Negl Trop Dis. 2019 Apr 25;13(4):e0007350. doi: 10.1371/journal.pntd.0007350 (PMC6504109; doi:10.1371/journal.pntd.0007350)
Supplement: S3 Table — (DOCX) [file pntd.0007350.s003.docx]

**S3 Table.** The matrix of correlation coefficients between all the selected variables on the grid scale.

|  | Bus stops | Subway stations | All stops | GDP | POP | Water | Vegetation | UVs | UL | NCL | CL | Road density | Incidence rates |
| --- | --- | --- | --- | --- | --- | --- | --- | --- | --- | --- | --- | --- | --- |
| Bus stops | 1.00 | 0.49 | 0.90 | 0.38 | 0.47 | -0.16 | -0.43 | 0.31 | -0.15 | 0.44 | 0.58 | 0.59 | 0.49 |
| Subway stations |  | 1.00 | 0.82 | 0.33 | 0.36 | -0.11 | -0.28 | 0.07 | -0.09 | 0.37 | 0.37 | 0.48 | 0.27 |
| All stops |  |  | 1.00 | 0.41 | 0.49 | -0.16 | -0.42 | 0.23 | -0.15 | 0.48 | 0.57 | 0.63 | 0.46 |
| GDP |  |  |  | 1.00 | 0.84 | -0.13 | -0.21 | 0.04 | -0.04 | 0.34 | 0.33 | 0.45 | 0.10 |
| POP |  |  |  |  | 1.00 | -0.09 | -0.31 | 0.17 | -0.19 | 0.40 | 0.46 | 0.51 | 0.17 |
| Water |  |  |  |  |  | 1.00 | -0.20 | -0.20 | -0.22 | -0.17 | -0.27 | -0.18 | -0.07 |
| Vegetation |  |  |  |  |  |  | 1.00 | -0.28 | 0.25 | -0.67 | -0.77 | -0.38 | -0.36 |
| UVs |  |  |  |  |  |  |  | 1.00 | -0.36 | -0.15 | 0.47 | 0.12 | 0.33 |
| UL |  |  |  |  |  |  |  |  | 1.00 | -0.02 | -0.24 | 0.00 | -0.24 |
| NCL |  |  |  |  |  |  |  |  |  | 1.00 | 0.80 | 0.56 | 0.26 |
| CL |  |  |  |  |  |  |  |  |  |  | 1.00 | 0.58 | 0.43 |
| Road density |  |  |  |  |  |  |  |  |  |  |  | 1.00 | 0.39 |
| Incidence rates |  |  |  |  |  |  |  |  |  |  |  |  | 1 |
| Abbreviations: GDP, POP, UVs, UL, NCL, and CL are respectively assigned for the gross domestic product, population density, urban villages, unused land, normal construction land, and construction land (both UVs and NCLs). | | | | | | | | | | | | | |
